# Supplementary material for: Mapping vaccine names in clinical trials to vaccine ontology using cascaded fine-tuned domain-specific language models
Source: J Biomed Semantics. 2024 Aug 10;15:14. doi: 10.1186/s13326-024-00318-x (PMC11316402; doi:10.1186/s13326-024-00318-x)
Supplement: Supplementary file 1 — Supplementary Material 1 [file 13326_2024_318_MOESM1_ESM.docx]

Table 1: Descriptions for error types

| Error Type | | Definition | Example | | |
| --- | --- | --- | --- | --- | --- |
|  |  |  | Mention | Gold concept | Predicted concept |
| NER | Multiple concepts | The gold concepts involve more than one concepts | diphtheria tetanus pertussis polio and haemophilus influenzae vaccine | diphtheria tetanus pertussis poliovirus vaccine\|haemophilus influenzae vaccine | diphtheria tetanus pertussis poliovirus haemophilus b vaccine |
|  | Mention with noise | Noise in the mention is unrelated to the gold concept. | dna hti 0 5ml at weeks 0 4 and 8 mva hti 0 5ml at weeks 12 and 20 dddmm | human immunodeficiency virus vaccine | mva |
| Abbreviation | | Abbreviations in the mention cause disambiguation errors. | chad63 mva me trap | plasmodium falciparum vaccine | mva |
| Disambiguation | | The mention contains multiple concepts, with the gold concept being one of them. However, the predicted concept corresponds to a different concept within the mention. | andes virus dna vaccine | andes virus vaccineusing adenovirus viral vector expressing andv np | dna vaccine |
| Hierarchy | Ancestor-grandchild | Gold and predicted concepts share ancestor-grandchild/parent-child/uncle-nephew/sibling relation in concept tree. | covid 19 vaccine hipra 10 | covid 19 subunit vaccine by hipra | covid 19 vaccine |
|  | Parent-child |  | bivalent recombinant covid 19 vaccine adenovirus type 5 vector for inhalation | covid 19 recombinant vector vaccine | covid 19 vaccine |
|  |  |  |  |  |  |
|  | Sibling |  | adjuvanted recombinant sars cov 2 trimerics protein subunit vaccine scb 2019 clover | scb 2019 | mf59 adjuvanted sars cov 2 sclamp vaccine |
| Semantic | | Gold and predicted concepts span more than two hierarchy levels. | coronavac covid 19 vaccine | coronavac | coronavirus vaccine |
| Stemming | | The mention is not tokenized correctly in concept normalization | boostrix poliotm | boostrix | boostrix polio |
| OOV | | The gold concept is not present in the ontology. | ad6nsmut mva nsmut | Not applicable | mva |

Box 1: SPARQL script to extract vaccine concepts from Ontobee

PREFIX owl: <http://www.w3.org/2002/07/owl#>

PREFIX rdfs: <http://www.w3.org/2000/01/rdf-schema#>

PREFIX obo: <http://purl.obolibrary.org/obo/>

SELECT DISTINCT ?Ontology_code ?Preferred_term ?Synonym

WHERE {

?Ontology_code rdfs:subClassOf* obo:VO_0000001 .

?Ontology_code rdfs:label ?Preferred_term .

OPTIONAL { ?Ontology_code obo:IAO_0000118 ?Synonym }

}
